# EARLY PANDEMIC INTERGENERATIONAL RELATIONSHIP TYPOLOGIES AND WELL-BEING AMONG OLDER ADULTS

**DOI:** 10.1093/geroni/igad104.1855

**Published:** 2023-12-21

**Authors:** Athena Chung Yin Chan, Rodlescia Sneed

**Affiliations:** Texas Tech University, Lubbock, Texas, United States; Wayne State University, Detroit, Michigan, United States

## Abstract

The COVID-19 pandemic necessitated numerous changes in older adults’ social interactions; however, there has been limited inquiry into the structure and function of early pandemic intergenerational relationships and consequent implications for well-being. Informed by intergenerational solidarity theory, we explored typologies of older adults’ intergenerational relationships early in the pandemic, examining factors associated with each typology and associations between typologies and well-being. Participants were 7,840 adults aged ≥ 50 from the 2020 and 2021 waves of the Health and Retirement Study. We conducted latent class analysis using 12 indicators: associational (time with pets, frequency of in-person and virtual contact), structural (pet ownership and living arrangement), functional (receiving/providing support), and affectual (better relations). We used analysis of variance and post hoc analyses to determine whether individual differences (i.e., demographics, health status, and pandemic-related stressors), and well-being differed across typologies. Five latent classes were identified: low intergenerational relations (48%), pet owner with low intergenerational relations (15%), high contact and good relationship quality (tightknit, 11%), support provider (15%) and support recipient (12%). Older adults’ age, gender, marital status, and health status were related to their relation typologies. Low intergenerational relations and tightknit typologies reported greater well-being. Support recipients reported the most distress, while support providers reported higher growth and resilience. Pet owners with low intergenerational relations reported the lowest positive emotions. Overall, results encourage careful attention to the multifaceted nature of intergenerational relationships when understanding coping of older adults in the pandemic.

